# Pathway to diagnosis and burden of illness in mucopolysaccharidosis type VII – a European caregiver survey

**DOI:** 10.1186/s13023-019-1233-z

**Published:** 2019-11-14

**Authors:** Alexandra Morrison, Esmee Oussoren, Tabea Friedel, Jordi Cruz, Nalan Yilmaz

**Affiliations:** 1 MPS Commercial, MPS House, Repton Place, White Lion Road, Amersham, HP7 9LP UK; 2 Center for Lysosomal and Metabolic Diseases, Erasmus MC, University Medical Center Rotterdam, P.O. Box 2060, 3000 CB Rotterdam, The Netherlands; 3 Gesellschaft für Mukopolysaccharidosen e.V, Herstallstrasse 35, 63739 Aschaffenburg, Germany; 4 Asociación MPS España, Anslem Clavé 1, 08787 La Pobla de Claramunt, Barcelona, Spain; 5 MPS LH Derneği, Hakimiyeti Milliye cad, No: 58 Vedat Kadri Kancal iş merkezi 46/A, Űskűdar, Istanbul, Turkey

**Keywords:** Mucopolysaccharidosis type VII, MPS VII, Sly disease, Lysosomal storage disorder, Burden of illness, Diagnostic delay, Caregiver burden, Diagnosis, Diagnostic odyssey

## Abstract

**Background:**

Mucopolysaccharidosis type VII (Sly disease, MPS VII), is an ultra-rare, multi-symptom disease with variable clinical presentations which can present challenges with diagnosis, management and care. We believe this survey is the first to explore the patient experience through direct questioning of the caregivers of 13 individuals with MPS VII.

**Methods:**

This European survey, using a specifically designed questionnaire, was conducted in order to describe the pathway to diagnosis and the burden of illness of MPS VII. Information on early symptoms, clinicians seen, and current symptoms was collected. Questions on the caregivers’ ability to work and the use and availability of health, social and educational support were included.

**Results:**

Caregivers of 13 patients from Germany, Spain, The Netherlands and Turkey responded to the survey. Five patients with non-immune hydrops fetalis (NIHF) were diagnosed with MPS VII at a mean age of 1.9 years (median 0.3 years, range 0.2 to 6 years). Those without NIHF (*n* = 7) were diagnosed at a mean age of 6.1 years (median 6.0 years, range 1.9 to 14 years). The symptoms most likely to raise a suspicion of MPS VII, excluding NIHF, did not appear until a median age of at least three years.

Over one half of patients required assistance with daily living and mobility. Reduction of the working hours of caregivers was often necessary (46.2% reduced hours, 30.8% stopped working).

Patients attended frequent medical appointments (12.7/year), over 80% had surgery and 30% had been hospitalised for respiratory issues.

While support for learning and behavioural needs was generally available, support for mobility was not available to 50% of patients. Half of the respondents (6/12) said they were not offered genetic counselling.

**Conclusions:**

For children that do not present with NIHF, diagnosis can take several years as early symptoms can be non-specific and mistaken for other conditions. Increased awareness of the early signs of disease and more information for parents/caregivers at diagnosis are needed. MPS VII poses significant burden to patients, caregivers, healthcare, social and educational services. Access to information and support varies across Europe and the availability of genetic counselling is limited in some countries.

## Background

Mucopolysaccharidosis type VII (Sly disease, MPS VII), is an ultra-rare autosomal recessive, lysosomal storage disorder caused by deficiency of the enzyme β-glucuronidase (*GUSB*). First described by Sly et al. in 1973, the absence of *GUSB* activity leads to the progressive accumulation of un-degraded glycosaminoglycans (GAGs) in many tissues of the body [1, 2]. The result is only partial degradation of the GAGs chondroitin, dermatan and heparan sulfate and leads to accumulation of these partially degraded fragments in the lysosomes of many tissues and organs, eventually leading to cellular and organ dysfunction [1]. In the United Kingdom, MPS VII is the rarest mucopolysaccharidosis with an average of only one affected birth every 10 years [3]. Worldwide it is estimated to have a frequency of less than 1:1,000,000 births [4].

The extreme rarity of MPS VII means that information on the natural history and clinical characteristics of the disease are scarce [5].

Patients can present with short stature, skeletal dysplasia, joint contractures, hepatosplenomegaly, hernias, cardiac involvement, pulmonary insufficiency, corneal clouding, hearing impairment and recurrent upper respiratory and middle ear infections [1, 6].

Cognitive, linguistic and social developmental delay is often present and patients may have behavioural disturbances such as hyperactivity, attention difficulties and extreme frustration [1, 7].

Affected patients can show a wide range of clinical variability, from early, severe, multisystem manifestations and progressive intellectual disability to a milder phenotype with later onset, fewer clinical manifestations and normal or near-normal intelligence [1, 6].

In the most severe cases, MPS VII presents as non-immune hydrops fetalis (NIHF) and may result in stillbirth or death within the first few weeks of life. However, the presence of NIHF does not always predict the severity of disease and does not always result in neonatal death [1].

Life expectancy is generally reduced due to frequent upper respiratory tract infections, neurodegenerative complications and abnormalities of the gastrointestinal tract, although patients with milder disease have survived into their fifth decade [1, 6].

As is the case with other rare diseases, patients with MPS VII may experience delays with their diagnosis, are often misdiagnosed and visit numerous doctors before they receive their final diagnosis [8]. In the absence of NIHF, MPS VII may not be suspected until several years after the symptoms have appeared. There is very little information on the evolution of symptoms, how patients are managed and under which specialty of clinicians prior to diagnosis. When there is a suspicion of MPS VII, the first step on the diagnostic pathway is through measurement of the GAGs dermatan, heparan and chondroitin sulfate in urine [9, 10]. GAG levels may be near normal in attenuated patients and therefore a test of enzyme activity in the blood is usually conducted when MPS VII is suspected [6]. Molecular genetic testing for mutations in the *GUSB* gene can be used to confirm the diagnosis [6]. Prenatal diagnosis is possible through amniocentesis to measure GAGs and *GUSB* activity or chorionic villus sampling for enzymatic and genetic analysis [11–14]. Genetic counselling is recommended for people with MPS VII and their families [3], yet it is not known if this is routinely available to all. Patients with MPS VII have significant and complex health needs. Current treatment options include haematopoietic stem cell transplantation (HSCT) with bone marrow or umbilical cord blood stem cells [3]. While experience of HSCT in MPS VII is limited, improvements in upper airway and respiratory function, hearing, vision, cardiac function, hepatosplenomegaly and joint mobility have been reported [15] and results suggest that it can slow or prevent further neurological complications [1, 15, 16].

Vestronidase alfa has received marketing approval in the US (2017) [17], Europe (2018) [18] and Brazil (2018) [19] as the first enzyme replacement therapy (ERT) for the treatment of non-neurological manifestations of MPS VII. In a recent Phase III trial, 12 patients with MPS VII were divided into 1 of 4 blinded groups with each group randomised to receive vestronidase alfa for 24 weeks at different time points. Treatment resulted in meaningful improvements to at least one of the clinical domains that the authors tested for that included a 6-min walk test, Forced Vital Capacity, shoulder flexion, visual acuity, and Bruininks-Oseretsky Test of Motor Proficiency (BOT-2) [20]. Symptomatic treatments of MPS VII include physiotherapy and hydrotherapy to improve activity levels, overall health and can help drain the build-up of mucus in the lungs [3]. Patients need regular clinical follow up and may require surgery to correct bone deformities, spinal cord compression, hernias, carpal tunnel syndrome and ocular and cardiovascular abnormalities [21, 22].

As new treatment options become available, the need for more information on the natural history and disease burden of MPS VII, to inform health economic analyses and health policy decisions on access to treatment becomes apparent [23]. Furthermore, consideration should be given to the non-healthcare related burden such as the loss of labour productivity for the caregiver. Through an increased understanding of the patient journey and burden of illness, suitable resources and support for patients and their families can be identified.

This survey aimed to support the recognition of the early symptoms of MPS VII by determining the pathway to diagnosis, to increase understanding of the burden of illness on patients, caregivers and healthcare resources and to determine the support that is needed by individuals with MPS VII and their families.

## Methods

### Study design and patient selection

The patient organisations and/or specialist clinicians in 25 European countries were approached to see if they were aware of any MPS VII patients. Patients and their parents/caregivers were invited to take part in the study by their local patient organisation or specialist clinician. The survey was also advertised in the MPS Society UK magazine and on their social media accounts.

### Inclusion criteria

Individuals or their parents/caregivers with MPS VII, living in Europe, were eligible to take part. In addition, the responder had to be ≥18 years, able to complete the questionnaire and to provide informed consent to participate.

### Assessments

The specifically designed questionnaire consisted of 59 questions arranged into the following sections: Person with MPS VII and their family, path to diagnosis, support for physical needs, support for medical needs, support for learning and behavioural needs and other support needed. The preferred method of completion was via face to face or telephone interview with a patient organisation that is local to the family. Where this was not possible, the option to fill the questionnaire out by post or e-mail was offered. Questionnaires were completed between 30 November 2017 and 31 March 2018.

## Results

### Demographics

A total of 18 patients were identified across 6 European countries. The parents/caregivers of 13 of these patients consented to take part in our study. One parent had two children with MPS VII, giving a total of 12 respondents. Five questionnaires were completed by telephone interview with the patient’s local patient organisation. Two were completed by the parent/caregiver with the help of their specialist clinician and the remaining six were completed by the parent/caregiver via post/e-mail.

The country of residence for patients were Germany (*n* = 2), Spain (*n* = 3), The Netherlands (*n* = 2) and Turkey (*n* = 6). The mean age of patients was 17.1 years (range 3.5 to 34 years). Five patients were currently receiving regular ERT and two patients had received a HSCT.

### Diagnosis in the presence or absence of NIHF

A diagnosis of NIHF was made in 38.5% of patients (5 out of 13), four patients before birth and one at birth or shortly after. Of the patients that presented with NIHF this led to testing and subsequent diagnosis of MPS VII in all but one patient. Patients with NIHF were generally diagnosed with MPS VII at an earlier age (mean 1.9 years, median 0.3 years, range 0.2 to 6 years, *n* = 5) than those without NIHF (mean 6.1 years, median 6.0 years, range 1.9 to 14 years, *n* = 7). For one individual diagnosed at birth, the presence or absence of NIHF was unknown by the responder. They noted that hospital investigations were undertaken due to the coarse facial features of the child. In those patients that did not present with NIHF (n = 7), the mean age when symptoms began was 1.4 years (median 0.3 years, range 0.1 to 5 years).

### Pathway to diagnosis where testing for MPS VII was not prompted by NIHF

**Fig. 1:**
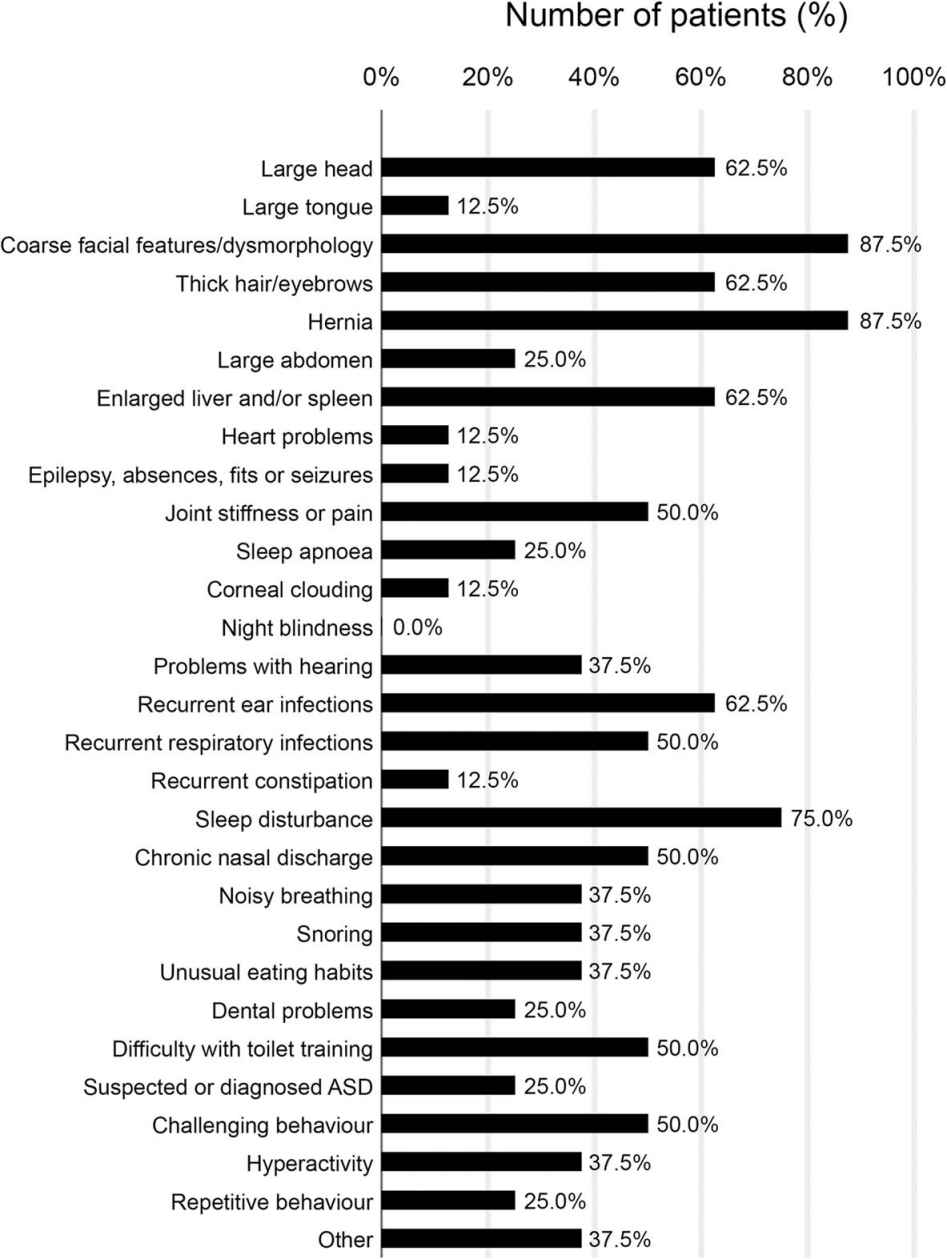
Symptoms present before diagnosis in patients not tested for MPS VII due to the presence of NIHF (*n* = 8*). This was a multiple-choice question. Respondents gave details of other symptoms for three patients: one patient with scoliosis, one patient with severe scoliosis, club foot and hip dislocation and one patient with cleft palate. *Symptoms for the patient diagnosed at birth in whom the NIHF status was unknown were not recorded by the respondent

For patients whose testing for MPS VII was not prompted by the presence of NIHF, respondents were asked to describe the symptoms that were present before diagnosis, who they consulted about symptoms and whether they had received any other diagnoses (no NIHF (*n* = 7), NIHF present (*n* = 1), NIHF status unknown (*n* = 1)). The most common symptoms before diagnosis were coarse features, hernias, sleep disturbance, recurrent ear infections, enlarged liver and/or spleen, thick hair/eyebrows and large head (Fig. 1). In those diagnosed over the age of two years (*n* = 7), delayed walking, speech and learning were common, occurring in 42.9% (3/7), 57.1% (4/7) and 57.1% (4/7), respectively. Loss of previously acquired walking ability, speech and learning prior to diagnosis was reported for 25.0% (2/8), 12.5% (1/8) and 12.5% (1/8) patients respectively.

**Fig. 2:**
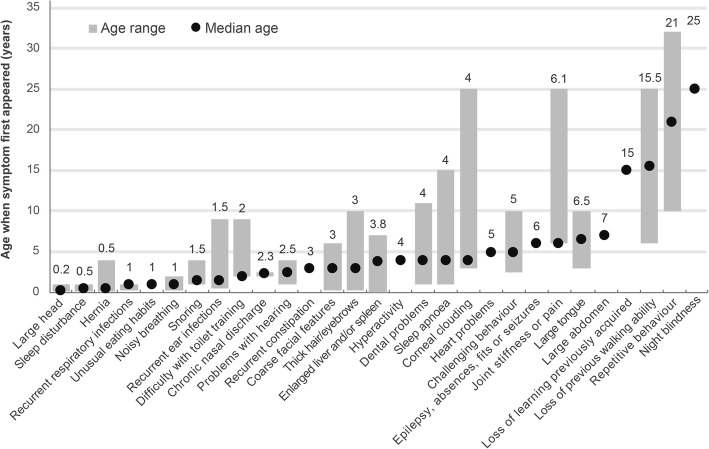
Age when symptoms first occurred in patients not tested for MPS VII due to the presence of NIHF (n = 8*). *Symptoms for the patient diagnosed at birth in whom the NIHF status was unknown were not recorded by the respondent. Respondents were asked to record the age of onset of pre-diagnosis symptoms only. However, five respondents recorded the age of onset for some symptoms as occurring after the age of diagnosis

Respondents were asked at what age the pre-diagnosis symptoms first appeared. Five respondents also gave us information on the appearance of symptoms after diagnosis and these are also reported in Fig. 2 to illustrate the later manifestations of disease. The earliest symptoms, appearing at a median age of under one year, were large head, sleep disturbance and hernia. Between the median ages of one and two years, recurrent respiratory infections, unusual eating habits, noisy breathing, snoring and recurrent ear infections were noted. By three years of age difficulty with toilet training, chronic nasal discharge and hearing problems were present. Children had recurrent constipation, coarse facial features, thick hair and eyebrows and an enlarged liver and/or spleen before their fourth birthday (Fig. 2).

**Fig. 3:**
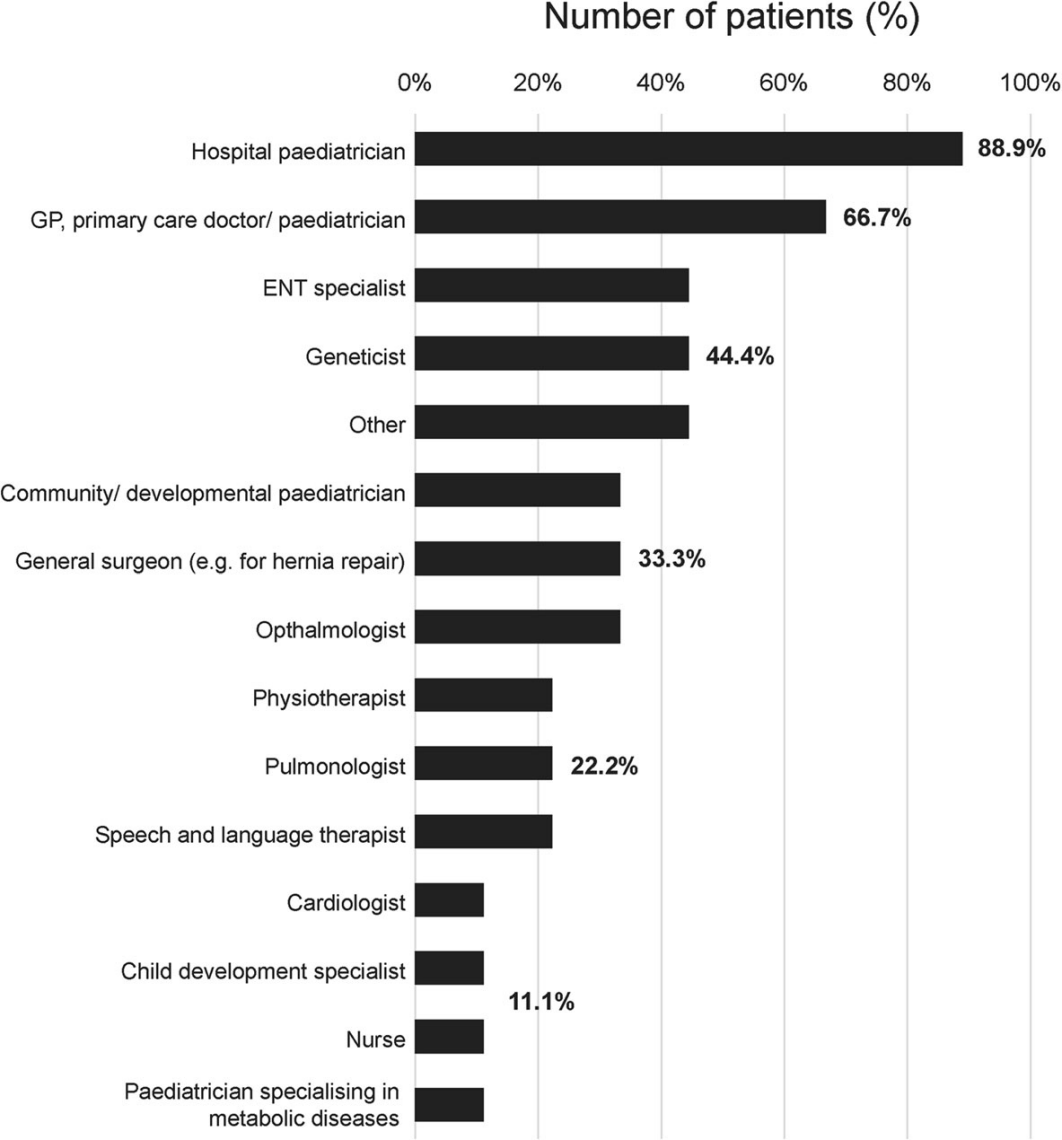
Healthcare professionals consulted before diagnosis of MPS VII by patients not tested for MPS VII due to the presence of NIHF (*n* = 9). This was a multiple-choice question, with the option to list any other healthcare professionals seen. Details of other healthcare professionals seen were noted for three patients: one patient was seen by a neurologist, traumatologist, psychologist, doctor of internal medicine and endocrinologist, one patient saw an orthopaedic doctor and one patient consulted a homeopath. ENT: ears, nose and throat

In most cases, patients were seen by more than one healthcare professional before their final diagnosis (mean 5.3 professionals), most commonly a hospital paediatrician (Fig. 3). Prior to the diagnosis of MPS VII, 25% (2/8) were diagnosed with autistic spectrum disorder (ASD) and 25% (2/8) with Perthes disease. One patient was diagnosed with attention deficit hyperactivity disorder (ADHD) and another with developmental delay.

The most common symptoms that lead to a suspicion of MPS were an enlarged liver and/or spleen (66.7%, 6/9), coarse facial features and thick hair/eyebrows (both 55.6%, 5/9). These features were not reported by parents/caregivers until a median age of at least three years, although some did present at an earlier age (Fig. 2). Joint stiffness or pain raised a suspicion of MPS VII in 44.4% of patients (4/9). This symptom was reported as occurring at a median age of 6.1 years (range 6 to 25 years). Delayed speech was another symptom leading to a suspicion of MPS VII in one third of patients (3/9). In one patient, MPS was not suspected although their GAGs were mildly elevated. Whole exome sequencing was performed due to their delayed cognitive development and identified the presence of MPS VII. This led to testing of their sibling who was also found to have MPS VII.

### Receiving a diagnosis

The diagnosis of MPS VII was made by a metabolic specialist consultant or a paediatrician specialising in metabolic diseases in most cases (61.5%, 8/13). The remainder were diagnosed by a geneticist (15.4%, 2/13), neurologist (15.4%, 2/13) or a paediatrician (7.7%, 1/13).

### Burden of illness

#### Patient burden

Overall, 61.5% (8/13) of patients were able to walk unaided, including two aged over 25 years. Four patients (30.8%, 4/13) were described as having lost walking ability previously acquired.

Use of walking aids was seen in 15.4% (2/13) of patients and 23.1% (3/13) required a wheelchair. The youngest patients to require walking aids or a wheelchair were in the age range of 16—20 years.

While 38.5% (5/13) of patients had age appropriate speech, almost half (46.2%, 6/13) had delayed speech development. In two of the adult patients (aged over 20 years) there was some deterioration of the speech that had previously been acquired. Six patients (6/12) spoke in short sentences, 16.7% (2/12) of patients could only use single words to communicate but could also use signs and hand signals.

Two patients (2/13) were described as having normal cognitive development for their age (one of these patients had received a HSCT). However, the majority of patients were described as having delayed cognitive development (69.2%, 9/13), while 15.4% (2/13) had lost some cognitive skills that had previously been acquired.

**Fig. 4:**
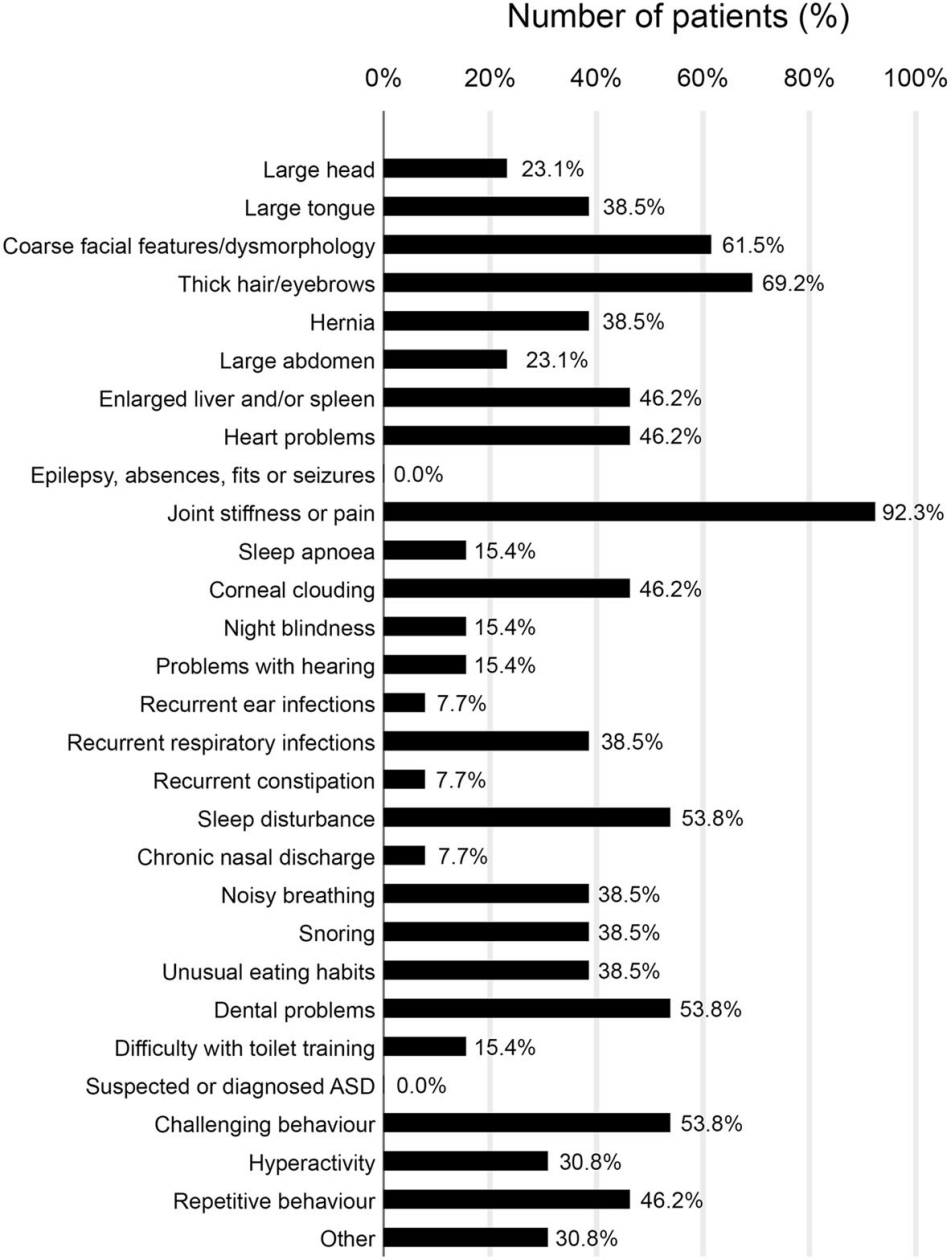
Current symptoms (*n* = 13). This was a multiple-choice question. Respondents gave details of other symptoms for four patients: one patient previously had difficulty with toilet training, one patient had severe scoliosis, short stature, a small and rigid thorax, hip dislocation and genu valgum, one patient had knee pain, back pain, side (spleen) pain and an enlarged spleen

Most patients (92.3%, 12/13) experienced joint stiffness or pain and thick hair and eyebrows (69.2%, 9/13). Coarse facial features (61.5%, 8/13) were common. Over half (53.8%, 7/13) were experiencing dental problems and sleep disturbance. Corneal clouding, enlarged liver and/or spleen and heart problems occurred in (46.2%, 6/13) of patients (Fig. 4).

Whilst 30.8% (4/13) of patients had no behavioural issues, most patients had some degree of behavioural symptoms such as short attention span, challenging behaviour or repetitive behaviour (all 46.2%, 6/13), hyperactivity (30.8%, 4/13) and no awareness of danger (7.7%, 1/13).

#### Caregiver burden

**Fig. 5:**
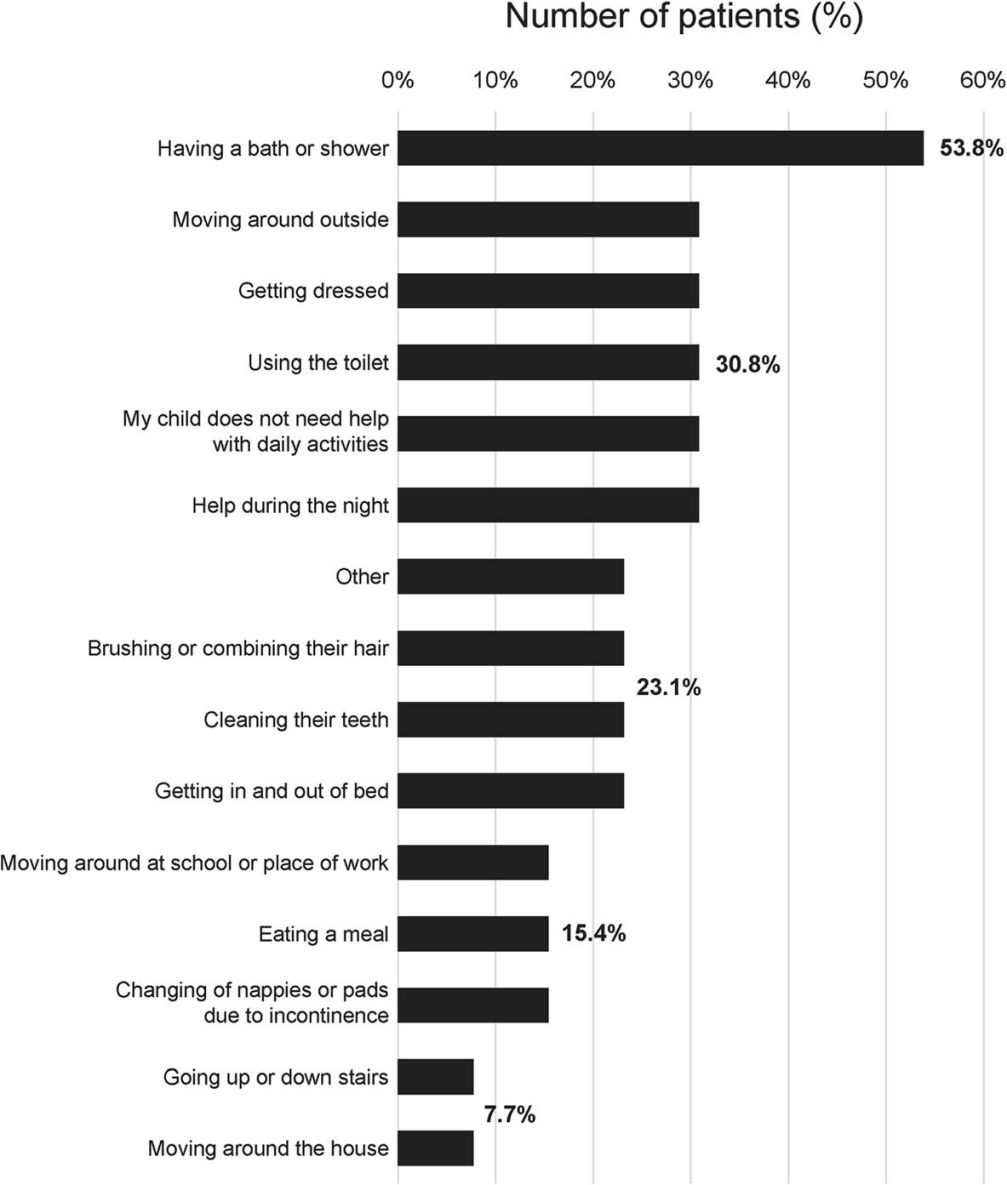
Help needed with daily activities (*n* = 13). Other help that was noted by respondents included family counselling, one-to-one to help in connection with their learning difficulties (to take pressure off mother), support in the morning to encourage independence, and assistance with wheelchair

When asked what the most difficult aspects of the disease to manage were, the most frequent answers related to behaviour and mobility. Just over 30% (4/13) of patients did not need any help with daily activities; but over half (53.8%, 7/13) required assistance with having a bath or shower and one third (30.8%, 4/13) required assistance with using the toilet, getting dressed, moving around outside and help during the night (Fig. 5). To adjust to issues with mobility, two families had to move to a more suitable house, one family installed a lift and two families said they had to provide other home adaptations for their child. Most patients were being cared for by their family, either by one (53.8%, 7/13) or two (30.8%, 4/13) parents, although 15.4% (2/13) were attended to by professional caregivers. Commonly, one parent either reduced their working hours (46.2%, 6/13) or stopped working (30.8%, 4/13) to care for their child. Over half (53.8%, 7/13) of patients have government provided financial support for care.

#### Medical needs

Most of the patients (84.6%, 11/13) had undergone at least one surgery, the most common was insertion of ear T-tubes or grommets. The other types of surgery or procedures undergone by patients were orthopaedic surgery, tonsillectomy, removal of adenoids, dental treatment under general anaesthesia and insertion of a shunt to treat hydrocephalus. The frequency and mean age at which patients had surgery is displayed in Table 1. Five patients were currently receiving ERT and most were on some other type of regular medication, the most common was for the heart (23.1%, 3/13), but only one patient was taking painkillers and one other was taking anti-inflammatories. More than half (58.3%, 7/12) were receiving physiotherapy and two patients were receiving animal therapy (hippotherapy). One third of patients (4/12) received no supportive therapies. These four patients were from Turkey and no requirement for additional therapies were identified by the parents/caregivers.

**Table 1:** Frequency and mean age of surgical procedures (*n* = 13)

Surgery	% patients (n/N)	Mean age (years)
Insertion of ear T-tubes or grommets	46.2, (6/13)	3.5
Removal of tonsils	30.8, (4/13)	3.1
Orthopaedic surgery (e.g. hip replacement)	30.8, (4/13)	11.5
Removal of adenoids	23.1, (3/13)	5.8
Dental treatment under general anaesthesia	23.1, (3/13)	9.0
Insertion of a shunt to treat hydrocephalus	7.7, (1/13)	2.5

**Fig. 6:**
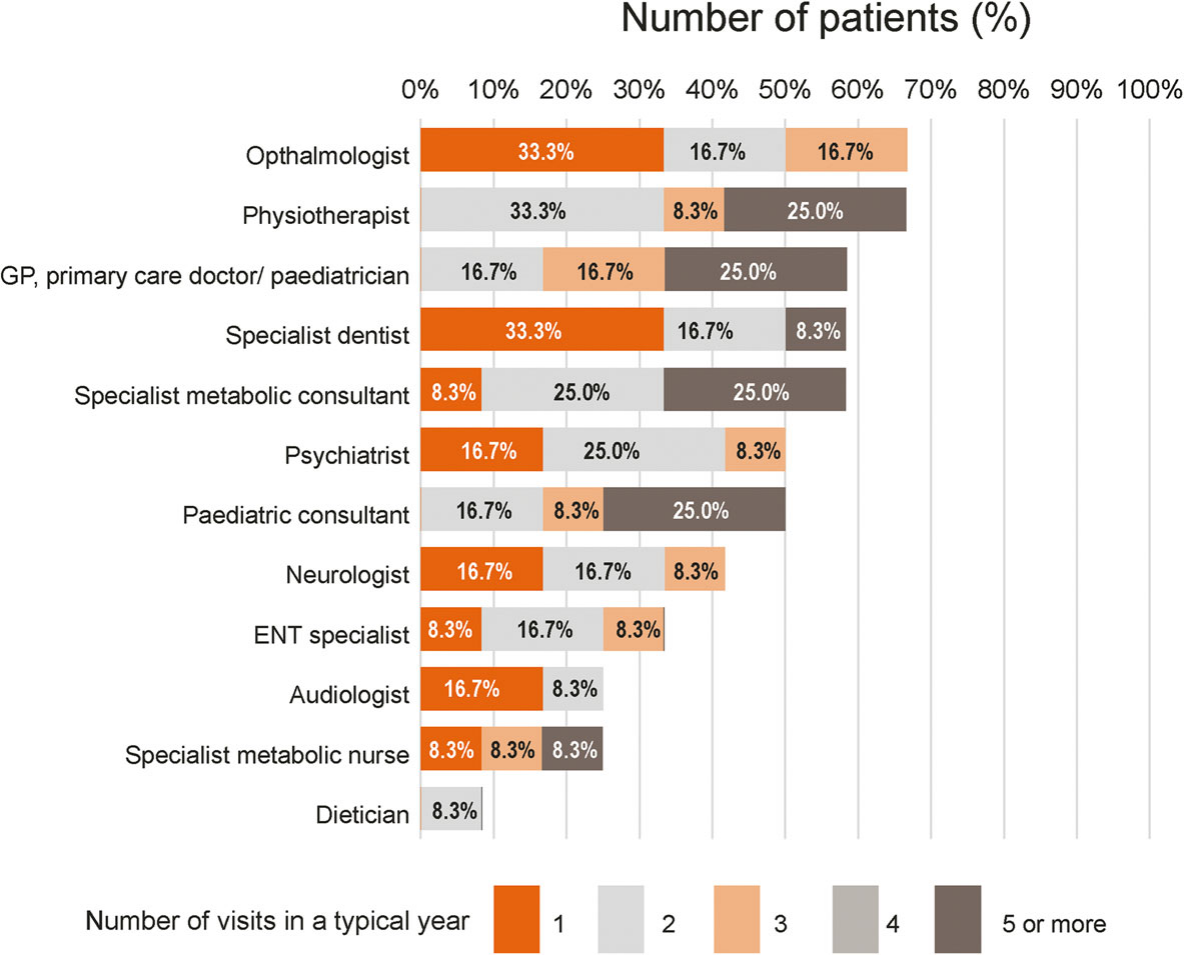
Number of medical appointments in a typical year (*n* = 12). Patients attend multiple appointments in a typical year and see a variety of medical professionals. This figure does not include visits made for a clinical trial or any additional comments that were listed by respondents in the questionnaire. These comments included ‘There are routine checks every six months’, ‘cardiologist once a year’, and ‘complete check-up when needed’. ENT: ears, nose and throat

Overall, patients would attend multiple appointments in a typical year (Fig. 6) with the total number of visits in a year ranging from 4 to 28 (mean 12.7, *n* = 12). Around 30% (4/13) of patients had been hospitalised for respiratory issues with patients staying in hospital for up to 10 days, although none had recorded time in intensive care.

### Learning and behavioural needs

Learning support was generally available where needed, although one parent/caregiver in Germany did not have access to support for their child’s learning.

Children most often started their education in a school for children of all abilities, with 41.7% (5/12) attending at least one specialist school for children with learning difficulties during their education. The mean age of children starting at a specialist school for children with learning difficulties was 7.6 years (range 1 to 14 years).

The most commonly provided school assistance was a specialist teacher, received by over half the patients at their first and second educational establishments (55.6% (5/9) and 60.0% (6/10) respectively). While no individuals received one-to-one support at their first educational establishment, 40% (4/10) received it at their second school. Professional support at school included that provided by physiotherapists, speech and language therapists, educational psychologists and special educational needs co-ordinators, received by 72.7% (8/11), 54.5% (6/11), 72.7% (8/11) and 54.5% (6/11) of patients respectively, at one or more of their educational establishments attended to date.

Sixty-nine percent (9/13) of patients had some behavioural issues. Most (67%, 6/9) were receiving support for their behavioural needs through their school. No respondents reported that this type of support was unavailable if needed.

### Support needs

After diagnosis, the information about the disease that was given to patients and their caregivers was variable with most receiving some information from a doctor, although two parents/caregivers received no information at all. Only respondents in Germany and The Netherlands were provided with information about a patient organisation and only one individual received any written information about the disease. Six out of 12 respondents (from Turkey (*n* = 4), Germany (*n* = 1) and Spain (*n* = 1)) were not offered genetic counselling. The individuals with MPS VII in these cases were diagnosed 15 or more years ago in Germany and Spain and between 3 and 14 years ago in Turkey. All but one of the respondents not offered genetic counselling would like access to this service.

When asked about the support that caregivers received for their child’s additional needs, only half (6/12) had access to support for their child’s mobility. Three respondents, from Turkey and Germany, did not have access to services to support home adaptations. Social care support such as social assistance and hospice/respite care was unavailable to 18% (2/11) of respondents, they were also residents of Turkey and Germany. Just over half (53.8%, 7/13) said they received government funded financial support for the care of their child.

## Discussion

This European survey is the first of its kind to describe MPS VII diagnosis capturing the patient and caregiver experience, their pathway to the final diagnosis of MPS VII, the burden of illness and the support that patients and their families need. Early diagnosis and appropriate treatment is likely to improve quality of life but for patients that do not present with NIHF, diagnosis can take several years. Early symptoms can be non-specific with variable clinical presentations and severity that can be mistaken for other conditions thus impeding rapid and accurate diagnosis. A key finding of this survey is that the symptoms that are most likely to raise suspicion of MPS VII, in the absence of NIHF, typically do not appear until age three or older. Enhancing disease awareness among healthcare professionals is paramount to ensure that the earliest symptoms can be identified and a suspicion of an MPS disorder raised for appropriate referral and further testing. Newer diagnostic techniques like whole exome sequencing can also help to identify MPS VII [24], as was the case for two patients in our study. This technique is particularly useful for patients in whom a genetic diagnosis is suspected, but the phenotype does not resemble a known syndrome or when biochemical and genetic testing have failed to arrive at a diagnosis [24].

In our survey, NIHF at birth was reported in approximately 40% of patients, a similar proportion to that reported in the literature [1], which is why it is important to include MPS VII in the differential diagnosis of NIHF. However, presentation of NIHF prenatally or at birth can result from a large number of different pathologies and identifying the cause depends on the thoroughness of testing to establish a diagnosis [25]. In a review of the literature to evaluate the incidence of lysosomal storage diseases in NIHF, the incidence varied between 1.3 to 5% of cases although of these cases MPS VII was the most common [26, 27]. MPS VII may account for as many as a quarter of the lysosomal storage disorders that present as NIHF [28]. Gimovsky et al. report that in 18–30% of NIHF cases the initial assessment revealed no probable cause but further testing resulted in a diagnosis of a lysosomal storage disorder in 17.4% of cases [26]. This highlights the need for comprehensive testing for lysosomal storage disorders following the initial assessment where NIHF is found to be idiopathic.

From this survey it was clear that even after diagnosis parents and caregivers are not always given adequate information about the condition. The information available at diagnosis varied by country, with some families in Spain and Turkey being given no information at all. This may be because medical professionals are not aware of the information that is available due to the rarity of the disease. Parents and caregivers find there is a lack of information about MPS VII, disease progression and treatment, highlighting both the need for increased awareness of the resources currently available and further research into the natural history and effects of treatment of MPS VII. A global, prospective, multicentre, longitudinal study called the Disease Monitoring Programme is currently underway. It is designed to characterise MPS VII disease presentation and progression over time in patients treated and not treated with ERT, and assess long-term effectiveness and safety of ERT, including hypersensitivity reactions and immunogenicity, in patients with MPS VII [29]. Clearly there is a need for such initiatives and the facility for patients, clinicians and researchers to record and share data on this ultra-rare disease.

The survey also highlights the need for greater access to genetic counselling with respondents in Turkey having the least access. Genetic counselling can help parents understand the medical implications, provide support and advice and an opportunity to discuss implications if further children are planned.

MPS VII imposes significant burden on patients and their families. Assessment of functional abilities in MPS patients show they are much lower than age-matched controls with an average score in the severe dependence range [23]. Parents/caregivers in our study identified issues with mobility as one of the most difficult aspects of the disease to manage. While the majority of patients (61.5%, 8/13) in our study could walk unaided, some adult patients needed walking aids or wheelchairs and most patients (69.2%, 9/13) needed help with daily activities. Péntek et al., reported that non-professional caregivers of patients with MPS spend a mean of 51 h per week giving informal care [23]. It is therefore not surprising that for 76.9% (10/13) of patients in this study, one parent/caregiver had reduced their working hours or stopped working. MPS VII can place a considerable financial burden on families, including loss of earnings and the need for adaptations to the home.

Parents/caregivers in this study found behavioural issues difficult to manage and reported behavioural difficulties in 69.2% (9/13) of patients. Previous studies of caregiver burden in MPS diseases have also reported behavioural symptoms to be among the most challenging for families along with sleep disturbance and communication difficulties [30, 31]. In our study, 53.8% (7/13) of patients currently had sleep disturbance, 30.8% (4/13) needed help during the night and only 50.0% (6/12) were described as speaking in an age appropriate manner. Dealing with these symptoms and the physical burden of daily care, can have a cumulative impact and impose significant psychological stress on caregivers [31].

While studies have shown that the quality of life of patients with MPS diseases and their caregivers is negatively impacted, there is a lack of specific measures for this patient population [32, 33]. The impact of MPS diseases on the family has been shown to be similar to that for other paediatric outpatients with chronic illnesses and caregiver burden increases with disease progression and loss of mobility [32].

Access to social support for mobility, care and housing was not necessarily available to all with some parents saying they would like more support. This survey and a previous survey of MPS patients and carers noted that health and social care varies across countries [23]. It is important not to under-estimate the importance of informal care and its impact on quality of life, both for patients and caregivers.

The costs to health services associated with MPS VII include treatment with disease modifying and supportive therapies, multiple medical appointments, hospitalisations and surgeries. The proportion of non-healthcare related formal costs, including the cost of professional caregivers, non-healthcare transport and social services should also be considered. Péntek et al. report that the majority of healthcare costs related to MPS are attributed to drugs, medical visits, hospitalisations and non-healthcare formal and informal care [23].

Many of the patients in our survey needed support in education. Generally, caregivers had access to additional support with learning and behavioural issues at school and/or via attendance at a specialist school for children with additional needs.

The main limitation of this study has been the small sample size, resulting from the extreme rarity of MPS VII. In addition, some respondents completing the questionnaire via post or e-mail skipped questions, leading to missing data. The heterogeneous population of patients and small numbers have limited any possibility of carrying out a rigorous statistical analysis of the data and resulted in a purely observational study. Furthermore, the most severe cases of MPS VII are not represented by this study as some MPS VII will go undiagnosed due to stillbirth, or death in the early weeks of life. As we only reported on patients who survived, the overall proportion of MPS VII patients who present with NIHF is likely to be higher than reported in our study.

## Conclusions

In this study we report that the pathway to a diagnosis of MPS VII can be lengthy even after the first symptoms have appeared and particularly where patients do not present with NIHF. Greater awareness of the early signs of MPS VII is needed and more understanding of the disease.

Parents and caregivers are not always provided with adequate information about the condition and may not be offered genetic counselling. Greater awareness of the resources available from patient organisations and specialist centres may be needed in some areas of Europe to support the clinicians giving a diagnosis of MPS VII and managing these patients.

This study highlights many aspects of disease burden for patients and their caregivers including the levels of care, housing, medical and educational support that are required. The burden of illness not only affects quality of life for patients but also for their caregivers, and there is an associated financial burden on families, healthcare, social and educational resources. Further studies are warranted to understand more fully the burden of illness and socio-economic impact of MPS VII in European and other countries around the world.

## Data Availability

The datasets collected and analysed during the current study are not publicly available for reasons of patient confidentiality.

## Abbreviations

ADHD: Attention deficit hyperactivity disorder
ASD: Autistic spectrum disorder
BOT-2: Bruininks-Oseretsky Test of Motor Proficiency
ENT: ear, nose and throat
ERT: Enzyme replacement therapy
GAG: Glycosaminoglycans
GP: General practitioner
*GUSB*: β-glucuronidase
HSCT: Haematopoietic stem cell transplantation
MPS VII: Mucopolysaccharidosis type VII
NIHF: Non-immune hydrops fetalis
